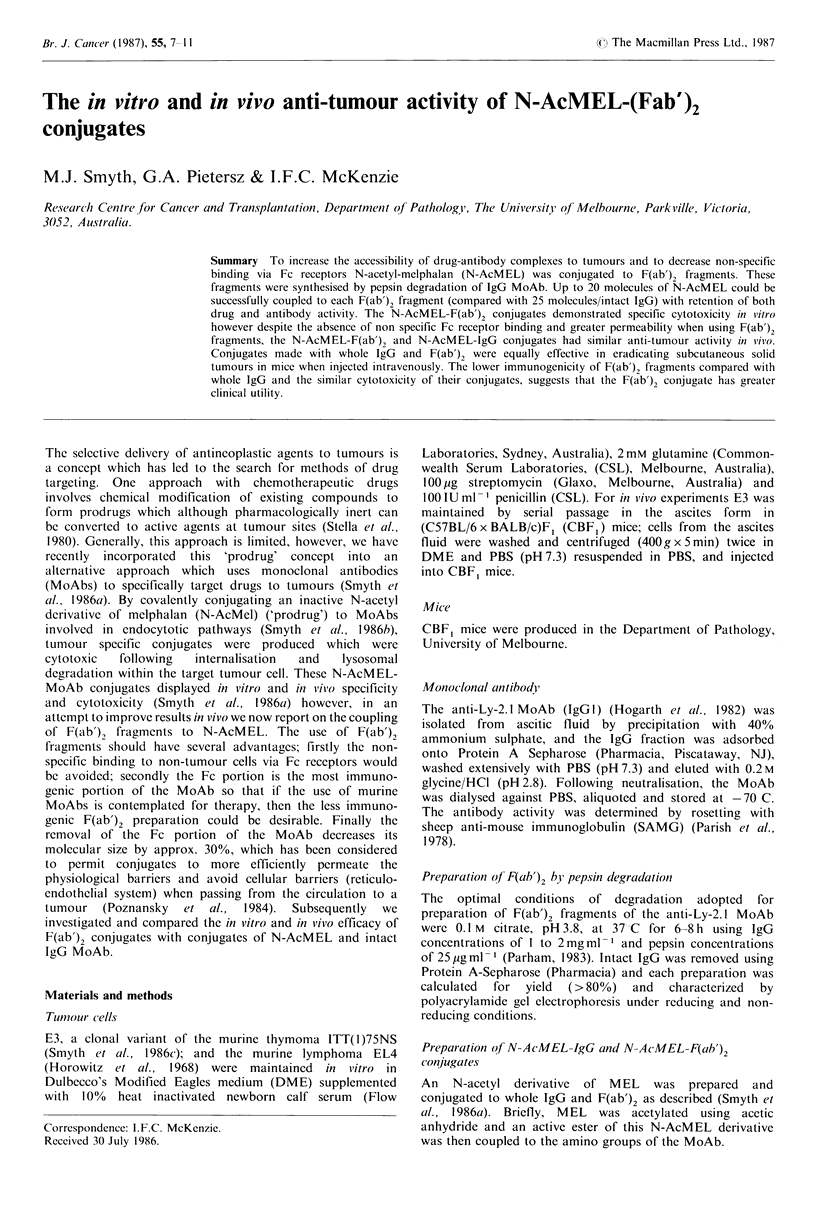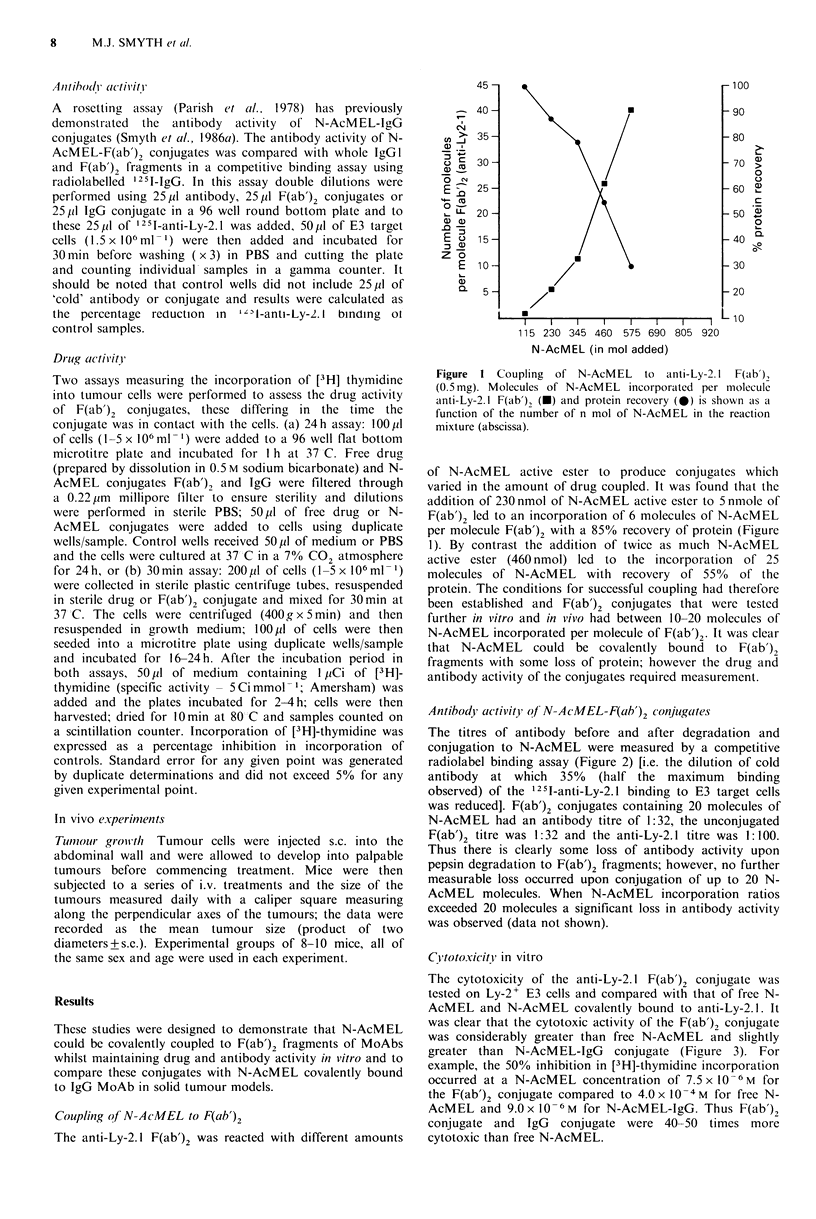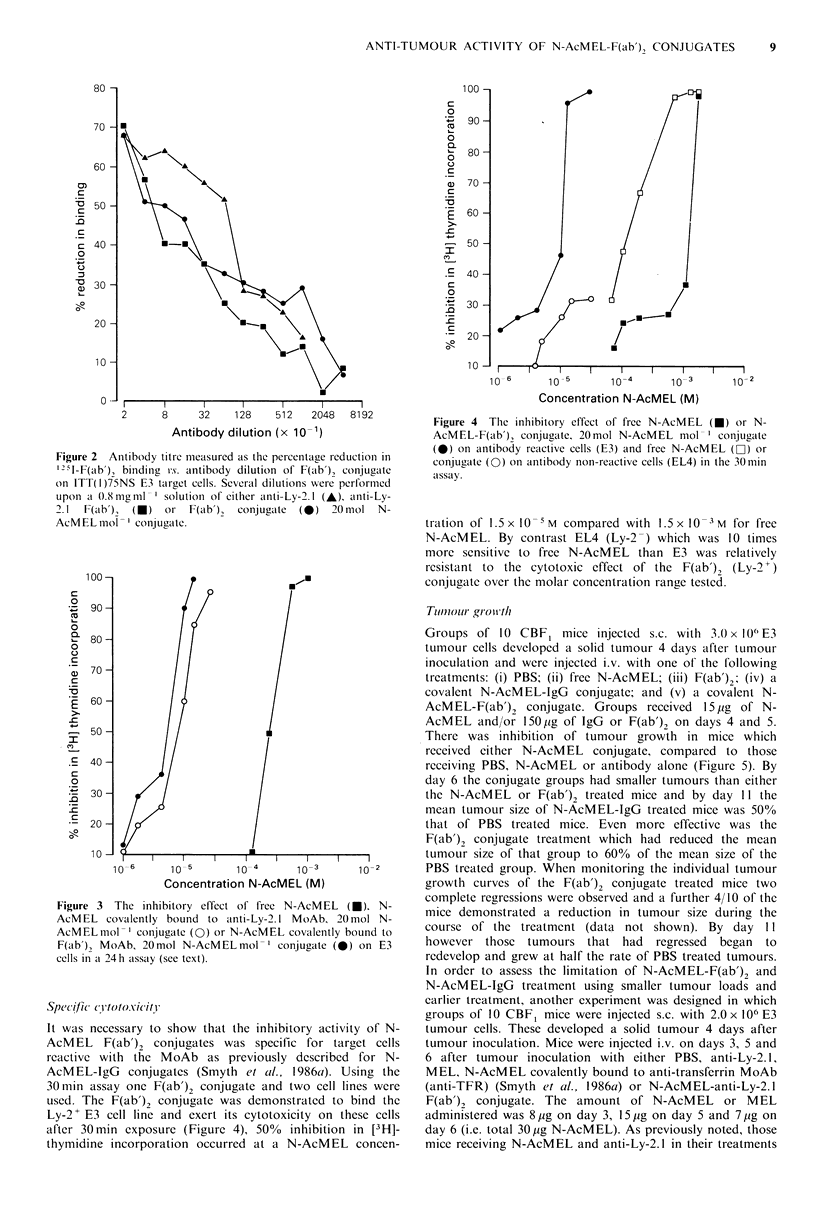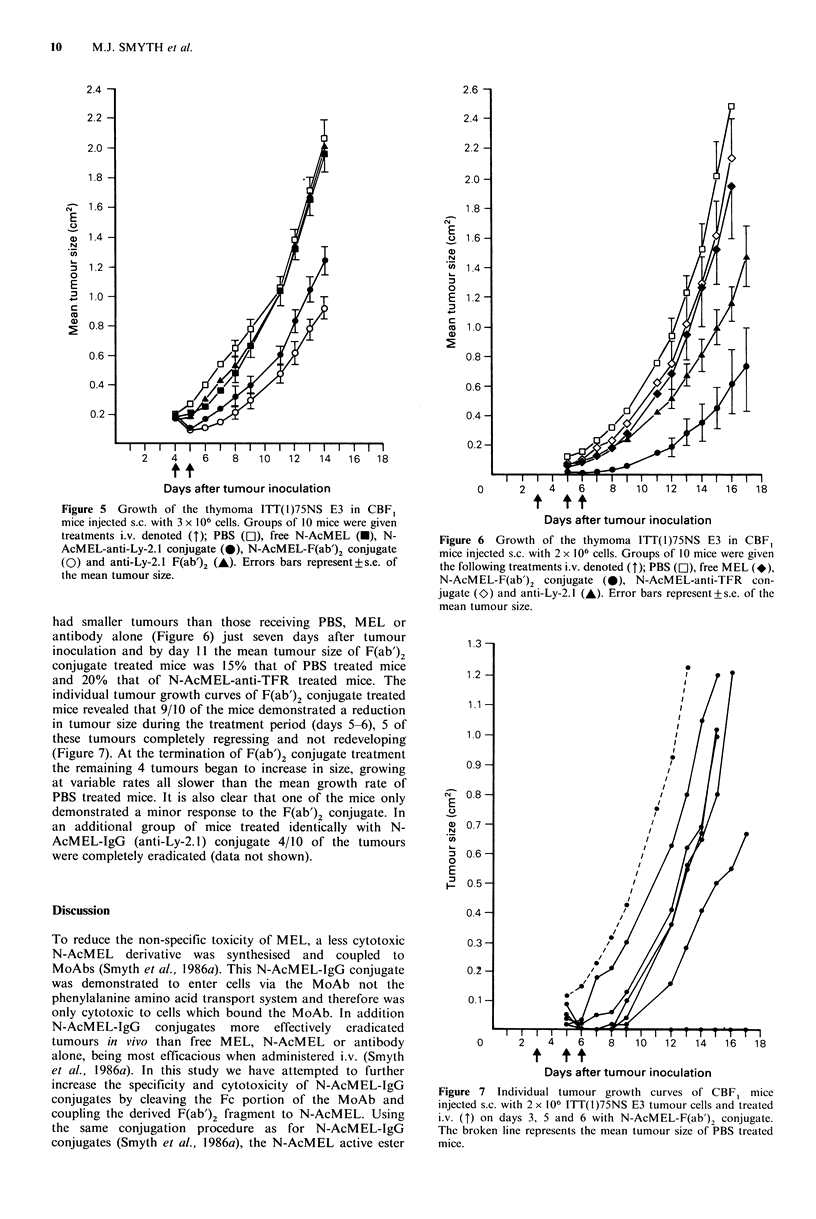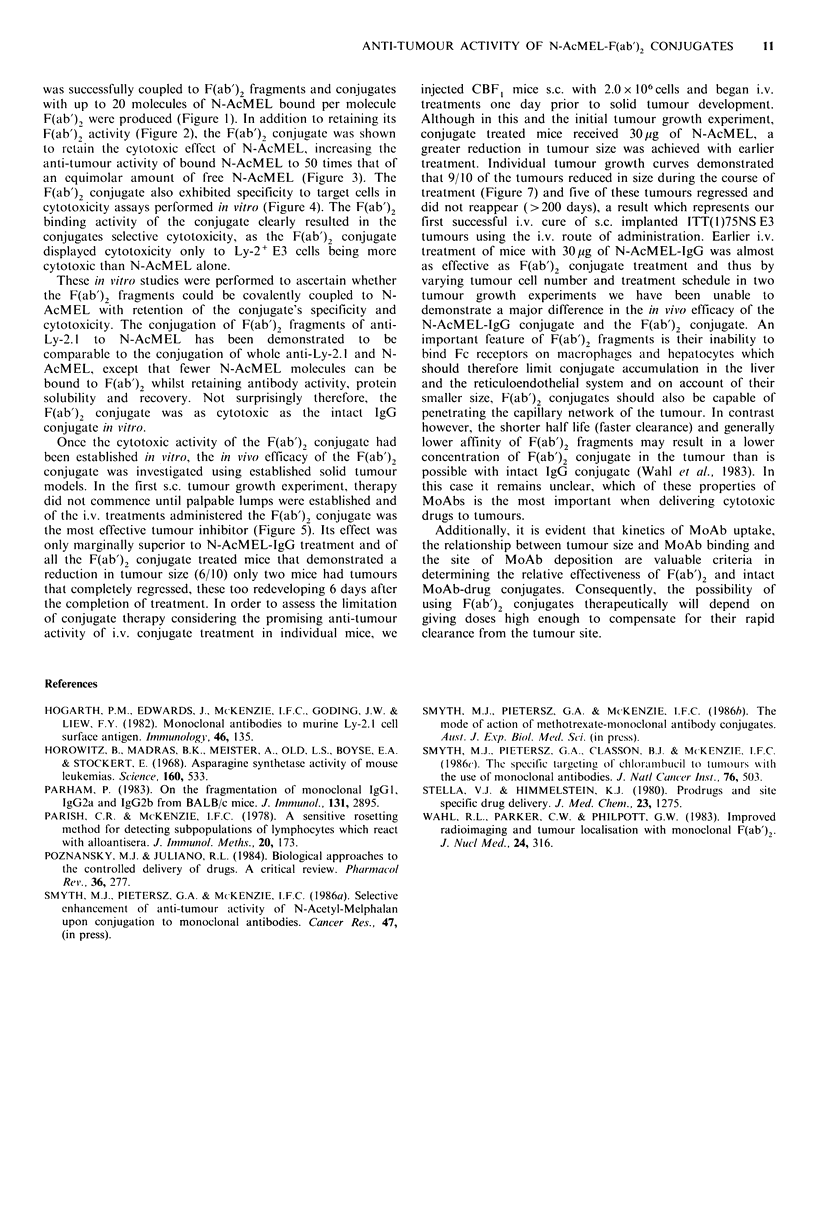# The in vitro and in vivo anti-tumour activity of N-AcMEL-(Fab')2 conjugates.

**DOI:** 10.1038/bjc.1987.2

**Published:** 1987-01

**Authors:** M. J. Smyth, G. A. Pietersz, I. F. McKenzie

## Abstract

To increase the accessibility of drug-antibody complexes to tumours and to decrease non-specific binding via Fc receptors N-acetyl-melphalan (N-AcMEL) was conjugated to F(ab')2 fragments. These fragments were synthesised by pepsin degradation of IgG MoAb. Up to 20 molecules of N-AcMEL could be successfully coupled to each F(ab')2 fragment (compared with 25 molecules/intact IgG) with retention of both drug and antibody activity. The N-AcMEL-F(ab')2 conjugates demonstrated specific cytotoxicity in vitro however despite the absence of non specific Fc receptor binding and greater permeability when using F(ab')2 fragments, the N-AcMEL-F(ab')2 and N-AcMEL-IgG conjugates had similar anti-tumour activity in vivo. Conjugates made with whole IgG and F(ab')2 were equally effective in eradicating subcutaneous solid tumours in mice when injected intravenously. The lower immunogenicity of F(ab')2 fragments compared with whole IgG and the similar cytotoxicity of their conjugates, suggests that the F(ab')2 conjugate has greater clinical utility.


*Br. J. Cancer* (1987), **55**, 7–11 © The Macmillan Press Ltd., 1987

### The *in vitro* and *in vivo* anti-tumour activity of N-AcMEL-(Fab′)$_2$ conjugates

M.J. Smyth, G.A. Pietersz & I.F.C. McKenzie

*Research Centre for Cancer and Transplantation, Department of Pathology, The University of Melbourne, Parkville, Victoria, 3052, Australia.*

**Summary** To increase the accessibility of drug-antibody complexes to tumours and to decrease non-specific binding via Fc receptors N-acetyl-melphalan (N-AcMEL) was conjugated to F(ab′)$_2$ fragments. These fragments were synthesised by pepsin degradation of IgG MoAb. Up to 20 molecules of N-AcMEL could be successfully coupled to each F(ab′)$_2$ fragment (compared with 25 molecules/intact IgG) with retention of both drug and antibody activity. The N-AcMEL-F(ab′)$_2$ conjugates demonstrated specific cytotoxicity *in vitro* however despite the absence of non specific Fc receptor binding and greater permeability when using F(ab′)$_2$ fragments, the N-AcMEL-F(ab′)$_2$ and N-AcMEL-IgG conjugates had similar anti-tumour activity *in vivo*. Conjugates made with whole IgG and F(ab′)$_2$ were equally effective in eradicating subcutaneous solid tumours in mice when injected intravenously. The lower immunogenicity of F(ab′)$_2$ fragments compared with whole IgG and the similar cytotoxicity of their conjugates, suggests that the F(ab′)$_2$ conjugate has greater clinical utility.

The selective delivery of antineoplastic agents to tumours is a concept which has led to the search for methods of drug targeting. One approach with chemotherapeutic drugs involves chemical modification of existing compounds to form prodrugs which although pharmacologically inert can be converted to active agents at tumour sites (Stella *et al.*, 1980). Generally, this approach is limited, however, we have recently incorporated this 'prodrug' concept into an alternative approach which uses monoclonal antibodies (MoAbs) to specifically target drugs to tumours (Smyth *et al.*, 1986*a*). By covalently conjugating an inactive N-acetyl derivative of melphalan (N-AcMel) ('prodrug') to MoAbs involved in endocytotic pathways (Smyth *et al.*, 1986*b*), tumour specific conjugates were produced which were cytotoxic following internalisation and lysosomal degradation within the target tumour cell. These N-AcMEL-MoAb conjugates displayed *in vitro* and *in vivo* specificity and cytotoxicity (Smyth *et al.*, 1986*a*) however, in an attempt to improve results *in vivo* we now report on the coupling of F(ab′)$_2$ fragments to N-AcMEL. The use of F(ab′)$_2$ fragments should have several advantages; firstly the non-specific binding to non-tumour cells via Fc receptors would be avoided; secondly the Fc portion is the most immunogenic portion of the MoAb so that if the use of murine MoAbs is contemplated for therapy, then the less immunogenic F(ab′)$_2$ preparation could be desirable. Finally the removal of the Fc portion of the MoAb decreases its molecular size by approx. 30%, which has been considered to permit conjugates to more efficiently permeate the physiological barriers and avoid cellular barriers (reticuloendothelial system) when passing from the circulation to a tumour (Poznansky *et al.*, 1984). Subsequently we investigated and compared the *in vitro* and *in vivo* efficacy of F(ab′)$_2$ conjugates with conjugates of N-AcMEL and intact IgG MoAb.

### Materials and methods

### *Tumour cells*

E3, a clonal variant of the murine thymoma ITT(1)75NS (Smyth *et al.*, 1986*c*); and the murine lymphoma EL4 (Horowitz *et al.*, 1968) were maintained *in vitro* in Dulbecco's Modified Eagles medium (DME) supplemented with 10% heat inactivated newborn calf serum (Flow Laboratories, Sydney, Australia), 2 mM glutamine (Commonwealth Serum Laboratories, (CSL), Melbourne, Australia), 100 $\mu$g streptomycin (Glaxo, Melbourne, Australia) and 100 IU ml$^{-1}$ penicillin (CSL). For *in vivo* experiments E3 was maintained by serial passage in the ascites form in (C57BL/6 × BALB/c)F$_1$ (CBF$_1$) mice; cells from the ascites fluid were washed and centrifuged (400 $g$ × 5 min) twice in DME and PBS (pH 7.3) resuspended in PBS, and injected into CBF$_1$ mice.

### *Mice*

CBF$_1$ mice were produced in the Department of Pathology, University of Melbourne.

### *Monoclonal antibody*

The anti-Ly-2.1 MoAb (IgG1) (Hogarth *et al.*, 1982) was isolated from ascitic fluid by precipitation with 40% ammonium sulphate, and the IgG fraction was adsorbed onto Protein A Sepharose (Pharmacia, Piscataway, NJ), washed extensively with PBS (pH 7.3) and eluted with 0.2 M glycine/HCl (pH 2.8). Following neutralisation, the MoAb was dialysed against PBS, aliquoted and stored at −70 C. The antibody activity was determined by rosetting with sheep anti-mouse immunoglobulin (SAMG) (Parish *et al.*, 1978).

### *Preparation of F(ab′)$_2$ by pepsin degradation*

The optimal conditions of degradation adopted for preparation of F(ab′)$_2$ fragments of the anti-Ly-2.1 MoAb were 0.1 M citrate, pH 3.8, at 37°C for 6–8 h using IgG concentrations of 1 to 2 mg ml$^{-1}$ and pepsin concentrations of 25 $\mu$g ml$^{-1}$ (Parham, 1983). Intact IgG was removed using Protein A-Sepharose (Pharmacia) and each preparation was calculated for yield (>80%) and characterized by polyacrylamide gel electrophoresis under reducing and non-reducing conditions.

### *Preparation of N-AcMEL-IgG and N-AcMEL-F(ab′)$_2$ conjugates*

An N-acetyl derivative of MEL was prepared and conjugated to whole IgG and F(ab′)$_2$ as described (Smyth *et al.*, 1986*a*). Briefly, MEL was acetylated using acetic anhydride and an active ester of this N-AcMEL derivative was then coupled to the amino groups of the MoAb.

Correspondence: I.F.C. McKenzie.
Received 30 July 1986.

*Antibody activity*

A rosetting assay (Parish *et al.*, 1978) has previously demonstrated the antibody activity of N-AcMEL-IgG conjugates (Smyth *et al.*, 1986*a*). The antibody activity of N-AcMEL-F(ab')$_2$ conjugates was compared with whole IgG1 and F(ab')$_2$ fragments in a competitive binding assay using radiolabelled $^{125}$I-IgG. In this assay double dilutions were performed using 25 $\mu$l antibody, 25 $\mu$l F(ab')$_2$ conjugates or 25 $\mu$l IgG conjugate in a 96 well round bottom plate and to these 25 $\mu$l of $^{125}$I-anti-Ly-2.1 was added, 50 $\mu$l of E3 target cells ($1.5\times10^6$ ml$^{-1}$) were then added and incubated for 30 min before washing ($\times 3$) in PBS and cutting the plate and counting individual samples in a gamma counter. It should be noted that control wells did not include 25 $\mu$l of 'cold' antibody or conjugate and results were calculated as the percentage reduction in $^{125}$I-anti-Ly-2.1 binding of control samples.

*Drug activity*

Two assays measuring the incorporation of [$^3$H] thymidine into tumour cells were performed to assess the drug activity of F(ab')$_2$ conjugates, these differing in the time the conjugate was in contact with the cells. (a) 24 h assay: 100 $\mu$l of cells ($1$–$5\times10^6$ ml$^{-1}$) were added to a 96 well flat bottom microtitre plate and incubated for 1 h at 37°C. Free drug (prepared by dissolution in 0.5 M sodium bicarbonate) and N-AcMEL conjugates F(ab')$_2$ and IgG were filtered through a 0.22 $\mu$m millipore filter to ensure sterility and dilutions were performed in sterile PBS; 50 $\mu$l of free drug or N-AcMEL conjugates were added to cells using duplicate wells/sample. Control wells received 50 $\mu$l of medium or PBS and the cells were cultured at 37°C in a 7% $CO_2$ atmosphere for 24 h, or (b) 30 min assay: 200 $\mu$l of cells ($1$–$5\times10^6$ ml$^{-1}$) were collected in sterile plastic centrifuge tubes, resuspended in sterile drug or F(ab')$_2$ conjugate and mixed for 30 min at 37°C. The cells were centrifuged ($400g\times5$ min) and then resuspended in growth medium; 100 $\mu$l of cells were then seeded into a microtitre plate using duplicate wells/sample and incubated for 16–24 h. After the incubation period in both assays, 50 $\mu$l of medium containing 1 $\mu$Ci of [$^3$H]-thymidine (specific activity – 5 Ci mmol$^{-1}$; Amersham) was added and the plates incubated for 2–4 h; cells were then harvested; dried for 10 min at 80°C and samples counted on a scintillation counter. Incorporation of [$^3$H]-thymidine was expressed as a percentage inhibition in incorporation of controls. Standard error for any given point was generated by duplicate determinations and did not exceed 5% for any given experimental point.

In vivo *experiments*

*Tumour growth* Tumour cells were injected s.c. into the abdominal wall and were allowed to develop into palpable tumours before commencing treatment. Mice were then subjected to a series of i.v. treatments and the size of the tumours measured daily with a caliper square measuring along the perpendicular axes of the tumours; the data were recorded as the mean tumour size (product of two diameters ± s.e.). Experimental groups of 8–10 mice, all of the same sex and age were used in each experiment.

### Results

These studies were designed to demonstrate that N-AcMEL could be covalently coupled to F(ab')$_2$ fragments of MoAbs whilst maintaining drug and antibody activity *in vitro* and to compare these conjugates with N-AcMEL covalently bound to IgG MoAb in solid tumour models.

*Coupling of N-AcMEL to F(ab')$_2$*

The anti-Ly-2.1 F(ab')$_2$ was reacted with different amounts of N-AcMEL active ester to produce conjugates which varied in the amount of drug coupled. It was found that the addition of 230 nmol of N-AcMEL active ester to 5 nmole of F(ab')$_2$ led to an incorporation of 6 molecules of N-AcMEL per molecule F(ab')$_2$ with a 85% recovery of protein (Figure 1). By contrast the addition of twice as much N-AcMEL active ester (460 nmol) led to the incorporation of 25 molecules of N-AcMEL with recovery of 55% of the protein. The conditions for successful coupling had therefore been established and F(ab')$_2$ conjugates that were tested further *in vitro* and *in vivo* had between 10–20 molecules of N-AcMEL incorporated per molecule of F(ab')$_2$. It was clear that N-AcMEL could be covalently bound to F(ab')$_2$ fragments with some loss of protein; however the drug and antibody activity of the conjugates required measurement.

**Figure 1** Coupling of N-AcMEL to anti-Ly-2.1 F(ab')$_2$ (0.5 mg). Molecules of N-AcMEL incorporated per molecule anti-Ly-2.1 F(ab')$_2$ (■) and protein recovery (●) is shown as a function of the number of n mol of N-AcMEL in the reaction mixture (abscissa).

*Antibody activity of N-AcMEL-F(ab')$_2$ conjugates*

The titres of antibody before and after degradation and conjugation to N-AcMEL were measured by a competitive radiolabel binding assay (Figure 2) [i.e. the dilution of cold antibody at which 35% (half the maximum binding observed) of the $^{125}$I-anti-Ly-2.1 binding to E3 target cells was reduced]. F(ab')$_2$ conjugates containing 20 molecules of N-AcMEL had an antibody titre of 1:32, the unconjugated F(ab')$_2$ titre was 1:32 and the anti-Ly-2.1 titre was 1:100. Thus there is clearly some loss of antibody activity upon pepsin degradation to F(ab')$_2$ fragments; however, no further measurable loss occurred upon conjugation of up to 20 N-AcMEL molecules. When N-AcMEL incorporation ratios exceeded 20 molecules a significant loss in antibody activity was observed (data not shown).

*Cytotoxicity* in vitro

The cytotoxicity of the anti-Ly-2.1 F(ab')$_2$ conjugate was tested on Ly-2$^+$ E3 cells and compared with that of free N-AcMEL and N-AcMEL covalently bound to anti-Ly-2.1. It was clear that the cytotoxic activity of the F(ab')$_2$ conjugate was considerably greater than free N-AcMEL and slightly greater than N-AcMEL-IgG conjugate (Figure 3). For example, the 50% inhibition in [$^3$H]-thymidine incorporation occurred at a N-AcMEL concentration of $7.5\times10^{-6}$ M for the F(ab')$_2$ conjugate compared to $4.0\times10^{-4}$ M for free N-AcMEL and $9.0\times10^{-6}$ M for N-AcMEL-IgG. Thus F(ab')$_2$ conjugate and IgG conjugate were 40–50 times more cytotoxic than free N-AcMEL.

**Figure 2** Antibody titre measured as the percentage reduction in $^{125}$I-F(ab')$_2$ binding *vs*. antibody dilution of F(ab')$_2$ conjugate on ITT(1)75NS E3 target cells. Several dilutions were performed upon a 0.8 mg ml$^{-1}$ solution of either anti-Ly-2.1 (▲), anti-Ly-2.1 F(ab')$_2$ (■) or F(ab')$_2$ conjugate (●) 20 mol N-AcMEL mol$^{-1}$ conjugate.

**Figure 3** The inhibitory effect of free N-AcMEL (■), N-AcMEL covalently bound to anti-Ly-2.1 MoAb, 20 mol N-AcMEL mol$^{-1}$ conjugate (○) or N-AcMEL covalently bound to F(ab')$_2$ MoAb, 20 mol N-AcMEL mol$^{-1}$ conjugate (●) on E3 cells in a 24 h assay (see text).

*Specific cytotoxicity*

It was necessary to show that the inhibitory activity of N-AcMEL F(ab')$_2$ conjugates was specific for target cells reactive with the MoAb as previously described for N-AcMEL-IgG conjugates (Smyth *et al.*, 1986*a*). Using the 30 min assay one F(ab')$_2$ conjugate and two cell lines were used. The F(ab')$_2$ conjugate was demonstrated to bind the Ly-2$^+$ E3 cell line and exert its cytotoxicity on these cells after 30 min exposure (Figure 4), 50% inhibition in [$^3$H]-thymidine incorporation occurred at a N-AcMEL concen-

**Figure 4** The inhibitory effect of free N-AcMEL (■) or N-AcMEL-F(ab')$_2$ conjugate, 20 mol N-AcMEL mol$^{-1}$ conjugate (●) on antibody reactive cells (E3) and free N-AcMEL (□) or conjugate (○) on antibody non-reactive cells (EL4) in the 30 min assay.

tration of $1.5 \times 10^{-5}$ M compared with $1.5 \times 10^{-3}$ M for free N-AcMEL. By contrast EL4 (Ly-2$^-$) which was 10 times more sensitive to free N-AcMEL than E3 was relatively resistant to the cytotoxic effect of the F(ab')$_2$ (Ly-2$^+$) conjugate over the molar concentration range tested.

*Tumour growth*

Groups of 10 CBF$_1$ mice injected s.c. with $3.0 \times 10^6$ E3 tumour cells developed a solid tumour 4 days after tumour inoculation and were injected i.v. with one of the following treatments: (i) PBS; (ii) free N-AcMEL; (iii) F(ab')$_2$; (iv) a covalent N-AcMEL-IgG conjugate; and (v) a covalent N-AcMEL-F(ab')$_2$ conjugate. Groups received 15 $\mu$g of N-AcMEL and/or 150 $\mu$g of IgG or F(ab')$_2$ on days 4 and 5. There was inhibition of tumour growth in mice which received either N-AcMEL conjugate, compared to those receiving PBS, N-AcMEL or antibody alone (Figure 5). By day 6 the conjugate groups had smaller tumours than either the N-AcMEL or F(ab')$_2$ treated mice and by day 11 the mean tumour size of N-AcMEL-IgG treated mice was 50% that of PBS treated mice. Even more effective was the F(ab')$_2$ conjugate treatment which had reduced the mean tumour size of that group to 60% of the mean size of the PBS treated group. When monitoring the individual tumour growth curves of the F(ab')$_2$ conjugate treated mice two complete regressions were observed and a further 4/10 of the mice demonstrated a reduction in tumour size during the course of the treatment (data not shown). By day 11 however those tumours that had regressed began to redevelop and grew at half the rate of PBS treated tumours. In order to assess the limitation of N-AcMEL-F(ab')$_2$ and N-AcMEL-IgG treatment using smaller tumour loads and earlier treatment, another experiment was designed in which groups of 10 CBF$_1$ mice were injected s.c. with $2.0 \times 10^6$ E3 tumour cells. These developed a solid tumour 4 days after tumour inoculation. Mice were injected i.v. on days 3, 5 and 6 after tumour inoculation with either PBS, anti-Ly-2.1, MEL, N-AcMEL covalently bound to anti-transferrin MoAb (anti-TFR) (Smyth *et al.*, 1986*a*) or N-AcMEL-anti-Ly-2.1 F(ab')$_2$ conjugate. The amount of N-AcMEL or MEL administered was 8 $\mu$g on day 3, 15 $\mu$g on day 5 and 7 $\mu$g on day 6 (i.e. total 30 $\mu$g N-AcMEL). As previously noted, those mice receiving N-AcMEL and anti-Ly-2.1 in their treatments

**Figure 5** Growth of the thymoma ITT(1)75NS E3 in $CBF_1$ mice injected s.c. with $3 \times 10^6$ cells. Groups of 10 mice were given treatments i.v. denoted (↑); PBS (□), free N-AcMEL (■), N-AcMEL-anti-Ly-2.1 conjugate (●), N-AcMEL-F(ab′)$_2$ conjugate (○) and anti-Ly-2.1 F(ab′)$_2$ (▲). Errors bars represent ± s.e. of the mean tumour size.

**Figure 6** Growth of the thymoma ITT(1)75NS E3 in $CBF_1$ mice injected s.c. with $2 \times 10^6$ cells. Groups of 10 mice were given the following treatments i.v. denoted (↑); PBS (□), free MEL (◆), N-AcMEL-F(ab′)$_2$ conjugate (●), N-AcMEL-anti-TFR conjugate (◇) and anti-Ly-2.1 (▲). Error bars represent ± s.e. of the mean tumour size.

had smaller tumours than those receiving PBS, MEL or antibody alone (Figure 6) just seven days after tumour inoculation and by day 11 the mean tumour size of F(ab′)$_2$ conjugate treated mice was 15% that of PBS treated mice and 20% that of N-AcMEL-anti-TFR treated mice. The individual tumour growth curves of F(ab′)$_2$ conjugate treated mice revealed that 9/10 of the mice demonstrated a reduction in tumour size during the treatment period (days 5–6), 5 of these tumours completely regressing and not redeveloping (Figure 7). At the termination of F(ab′)$_2$ conjugate treatment the remaining 4 tumours began to increase in size, growing at variable rates all slower than the mean growth rate of PBS treated mice. It is also clear that one of the mice only demonstrated a minor response to the F(ab′)$_2$ conjugate. In an additional group of mice treated identically with N-AcMEL-IgG (anti-Ly-2.1) conjugate 4/10 of the tumours were completely eradicated (data not shown).

### Discussion

To reduce the non-specific toxicity of MEL, a less cytotoxic N-AcMEL derivative was synthesised and coupled to MoAbs (Smyth *et al.*, 1986*a*). This N-AcMEL-IgG conjugate was demonstrated to enter cells via the MoAb not the phenylalanine amino acid transport system and therefore was only cytotoxic to cells which bound the MoAb. In addition N-AcMEL-IgG conjugates more effectively eradicated tumours *in vivo* than free MEL, N-AcMEL or antibody alone, being most efficacious when administered i.v. (Smyth *et al.*, 1986*a*). In this study we have attempted to further increase the specificity and cytotoxicity of N-AcMEL-IgG conjugates by cleaving the Fc portion of the MoAb and coupling the derived F(ab′)$_2$ fragment to N-AcMEL. Using the same conjugation procedure as for N-AcMEL-IgG conjugates (Smyth *et al.*, 1986*a*), the N-AcMEL active ester

**Figure 7** Individual tumour growth curves of $CBF_1$ mice injected s.c. with $2 \times 10^6$ ITT(1)75NS E3 tumour cells and treated i.v. (↑) on days 3, 5 and 6 with N-AcMEL-F(ab′)$_2$ conjugate. The broken line represents the mean tumour size of PBS treated mice.

was successfully coupled to F(ab')$_2$ fragments and conjugates with up to 20 molecules of N-AcMEL bound per molecule F(ab')$_2$ were produced (Figure 1). In addition to retaining its F(ab')$_2$ activity (Figure 2), the F(ab')$_2$ conjugate was shown to retain the cytotoxic effect of N-AcMEL, increasing the anti-tumour activity of bound N-AcMEL to 50 times that of an equimolar amount of free N-AcMEL (Figure 3). The F(ab')$_2$ conjugate also exhibited specificity to target cells in cytotoxicity assays performed *in vitro* (Figure 4). The F(ab')$_2$ binding activity of the conjugate clearly resulted in the conjugates selective cytotoxicity, as the F(ab')$_2$ conjugate displayed cytotoxicity only to Ly-2$^+$ E3 cells being more cytotoxic than N-AcMEL alone.

These *in vitro* studies were performed to ascertain whether the F(ab')$_2$ fragments could be covalently coupled to N-AcMEL with retention of the conjugate's specificity and cytotoxicity. The conjugation of F(ab')$_2$ fragments of anti-Ly-2.1 to N-AcMEL has been demonstrated to be comparable to the conjugation of whole anti-Ly-2.1 and N-AcMEL, except that fewer N-AcMEL molecules can be bound to F(ab')$_2$ whilst retaining antibody activity, protein solubility and recovery. Not surprisingly therefore, the F(ab')$_2$ conjugate was as cytotoxic as the intact IgG conjugate *in vitro*.

Once the cytotoxic activity of the F(ab')$_2$ conjugate had been established *in vitro*, the *in vivo* efficacy of the F(ab')$_2$ conjugate was investigated using established solid tumour models. In the first s.c. tumour growth experiment, therapy did not commence until palpable lumps were established and of the i.v. treatments administered the F(ab')$_2$ conjugate was the most effective tumour inhibitor (Figure 5). Its effect was only marginally superior to N-AcMEL-IgG treatment and of all the F(ab')$_2$ conjugate treated mice that demonstrated a reduction in tumour size (6/10) only two mice had tumours that completely regressed, these too redeveloping 6 days after the completion of treatment. In order to assess the limitation of conjugate therapy considering the promising anti-tumour activity of i.v. conjugate treatment in individual mice, we injected CBF$_1$ mice s.c. with $2.0 \times 10^6$ cells and began i.v. treatments one day prior to solid tumour development. Although in this and the initial tumour growth experiment, conjugate treated mice received 30 $\mu g$ of N-AcMEL, a greater reduction in tumour size was achieved with earlier treatment. Individual tumour growth curves demonstrated that 9/10 of the tumours reduced in size during the course of treatment (Figure 7) and five of these tumours regressed and did not reappear (>200 days), a result which represents our first successful i.v. cure of s.c. implanted ITT(1)75NS E3 tumours using the i.v. route of administration. Earlier i.v. treatment of mice with 30 $\mu g$ of N-AcMEL-IgG was almost as effective as F(ab')$_2$ conjugate treatment and thus by varying tumour cell number and treatment schedule in two tumour growth experiments we have been unable to demonstrate a major difference in the *in vivo* efficacy of the N-AcMEL-IgG conjugate and the F(ab')$_2$ conjugate. An important feature of F(ab')$_2$ fragments is their inability to bind Fc receptors on macrophages and hepatocytes which should therefore limit conjugate accumulation in the liver and the reticuloendothelial system and on account of their smaller size, F(ab')$_2$ conjugates should also be capable of penetrating the capillary network of the tumour. In contrast however, the shorter half life (faster clearance) and generally lower affinity of F(ab')$_2$ fragments may result in a lower concentration of F(ab')$_2$ conjugate in the tumour than is possible with intact IgG conjugate (Wahl *et al.*, 1983). In this case it remains unclear, which of these properties of MoAbs is the most important when delivering cytotoxic drugs to tumours.

Additionally, it is evident that kinetics of MoAb uptake, the relationship between tumour size and MoAb binding and the site of MoAb deposition are valuable criteria in determining the relative effectiveness of F(ab')$_2$ and intact MoAb-drug conjugates. Consequently, the possibility of using F(ab')$_2$ conjugates therapeutically will depend on giving doses high enough to compensate for their rapid clearance from the tumour site.

### References

HOGARTH, P.M., EDWARDS, J., McKENZIE, I.F.C., GODING, J.W. & LIEW, F.Y. (1982). Monoclonal antibodies to murine Ly-2.1 cell surface antigen. *Immunology*, **46**, 135.

HOROWITZ, B., MADRAS, B.K., MEISTER, A., OLD, L.S., BOYSE, E.A. & STOCKERT, E. (1968). Asparagine synthetase activity of mouse leukemias. *Science*, **160**, 533.

PARHAM, P. (1983). On the fragmentation of monoclonal IgG1, IgG2a and IgG2b from BALB/c mice. *J. Immunol.*, **131**, 2895.

PARISH, C.R. & McKENZIE, I.F.C. (1978). A sensitive rosetting method for detecting subpopulations of lymphocytes which react with alloantisera. *J. Immunol. Meths.*, **20**, 173.

POZNANSKY, M.J. & JULIANO, R.L. (1984). Biological approaches to the controlled delivery of drugs. A critical review. *Pharmacol Rev.*, **36**, 277.

SMYTH, M.J., PIETERSZ, G.A. & McKENZIE, I.F.C. (1986*a*). Selective enhancement of anti-tumour activity of N-Acetyl-Melphalan upon conjugation to monoclonal antibodies. *Cancer Res.*, **47**, (in press).

SMYTH, M.J., PIETERSZ, G.A. & McKENZIE, I.F.C. (1986*b*). The mode of action of methotrexate-monoclonal antibody conjugates. *Aust. J. Exp. Biol. Med. Sci.* (in press).

SMYTH, M.J., PIETERSZ, G.A., CLASSON, B.J. & McKENZIE, I.F.C. (1986*c*). The specific targeting of chlorambucil to tumours with the use of monoclonal antibodies. *J. Natl Cancer Inst.*, **76**, 503.

STELLA, V.J. & HIMMELSTEIN, K.J. (1980). Prodrugs and site specific drug delivery. *J. Med. Chem.*, **23**, 1275.

WAHL, R.L., PARKER, C.W. & PHILPOTT, G.W. (1983). Improved radioimaging and tumour localisation with monoclonal F(ab')$_2$. *J. Nucl Med.*, **24**, 316.